# Association rickettsiose et infarctus cérébral: une nouvelle observation

**DOI:** 10.11604/pamj.2017.26.80.11434

**Published:** 2017-02-20

**Authors:** Tarik Boulahri, Abdellah Taous, Maha Aït Berri, Imane Traibi, Jalal Elbenaye, Abdelhadi Rouimi

**Affiliations:** 1 Service de Neurlogie, Hopital Militaire Moulay Ismail, Meknès, Maroc; 2 Service de Dermatologie, Hopital Militaire Moulay Ismail Meknès, Maroc

**Keywords:** Rickettsiose, infarctus cérébral, IRM cérébrale, Rickettsiosis, cerebral infarction, MRI of the brain

## Abstract

La fièvre boutonneuse méditerranéenne (FBM) est une rickettsiose du groupe boutonneux due à rickettsia conorii. Cette zoonose est réputée d'évolution bénigne mais peut se compliquer dans les formes sévères d'une atteinte neurologique qui en font parfois toute la gravité. Nous rapportons l'observation d'une patiente âgée de 49 ans ayant présenté au cours de son hospitalisation en dermatologie pour une rickettsiose, une hémiplégie droite massive d'installation brutale. L'angio-IRM cérébrale a objectivé un accident vasculaire cérébral ischémique sylvien profond gauche. Le diagnostic de rickettsiose de type conorii a été retenu sur l'aspect des lésions dermatologiques et la positivité des sérologies par immuno-fluorescence indirecte. L'évolution était favorable sous anti-biothérapie (doxycycline et fluoroquinolone). Exceptionnellement rapporté dans le cadre des manifestations neurologiques des rickettioses, l'infarctus cérébral reste une complication à ne pas méconnaitre surtout en regard d'un bilan étiologique en particulier cardiovasculaire demeuré négatif.

## Abstract (en)

Mediterranean spotted fever (MSF) is a rickettsiosis of the spotted fever group caused by rickettsia conorii. This zoonosis is benign but it can be complicated by severe neurological impairment (hence its severity). We report the case of a 49-year old patient hospitalized in the Department of Dermatology for rickettsiosis, who suffered from right massive hemiplegia of brutal onset. Magnetic resonance angiography (MRA) of the brain showed deep left sylvian fissure ischemic stroke. The diagnosis of conorii-type rickettsiosis has been retained based on the aspect of skin lesions and on positive sierologic testing with the indirect immunofluorescence method. Evolution was favorable under anti-biotherapy (Doxycycline and fluoroquinolone). Cerebral infarction, exceptionally reported in the framework of neurological manifestations of rickettsioses, is a complication that shouldn’t be underestimated especially when etiologic assessment (cardiovascular in particular) is negative.

## Introduction

La fièvre boutonneuse méditerranéenne (FBM) est une rickettsiose du groupe boutonneux due à rickettsia conorii. Cette zoonose est réputée d'évolution bénigne mais peut se compliquer dans les formes sévères d'une atteinte neurologique qui en font parfois toute la gravité. Nous rapportons un cas d'accident vasculaire cérébral ischémique (AVCI) dû à une rickettsiose.

## Patient et observation

**Figure 1:**
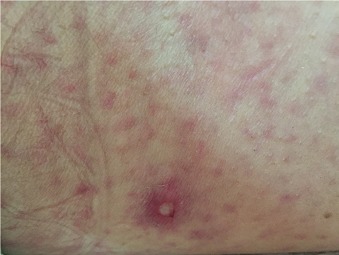
Eruption maculo-papuleuse purpurique diffuse avec escarre d’inoculation au flanc gauche

**Figure 2:**
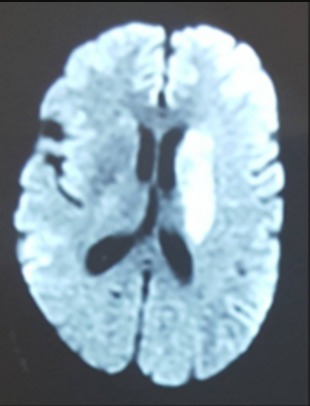
IRM cérébrale coupe axiale en séquence diffusion montrant un hypersignal capsulo-lenticulo-caudé gauche compatible avec un AVCI sylvien profond gauchequa

Il s'agit d'une patiente âgée de 50 ans, sans facteur de risque cardiovasculaire, ayant présenté au 5^ème^ jour de son hospitalisation en dermatologie pour rickettsiose une lourdeur de l'hémicorps droit avec une suspension du langage d'installation brutale. L'examen général a trouvé une patiente consciente, aphasique, fébrile à 39°C, une tension artérielle à 180/90 mmHg avec une nuque souple. L'examen neurologique a révélé une hémiplégie droite flasque massive, une paralysie faciale centrale droite, une hémianopsie latérale homonyme et une aphasie non fluente. Son score NIHSS initial était à 19. L'examen cutanéo-muqueux a montré une éruption maculo-papuleuse purpurique diffuse avec escarre d'inoculation au flanc gauche (Figure 1). L'examen cardio-vasculaire était sans anomalie. La TDM cérébrale, réalisée à H1, était normale. L'Angio-IRM cérébrale a montré un AVCI sylvien profond gauche (Figure 2). Un bilan biologique a révélé une VS à 40mm à la première heure, une CRP à 25 mg/l, une thrombopénie à 110000/mm^3^ et une anémie inflammatoire à 10 g/dl. L'ionogramme sanguin, les bilans hépatique et rénal étaient normaux. Les sérologies virales (hépatites B et C, VIH) et syphilitique étaient négatives. Le bilan cardio-vasculaire (ECG, ETT, ETO, Echo-doppler des vaisseaux du cou, Holter ECG) était normal de même que l'étude cyto-chimique du liquide céphalo-rachidien. Le diagnostic de rickettsiose de type conorii a été retenu sur l'aspect des lésions dermatologiques et la positivité des sérologies par immuno-fluorescence indirecte. Au terme de ce bilan, l'AVCI a été imputé à la rickettsiose et la patiente a été mise sous bi-antibiothérapie associant la Doxycycline et la Ciprofloxacine, pendant dix jours, avec une nette amélioration clinique après 4 semaines.

## Discussion

La FBM est une maladie due à une bactérie intracellulaire obligatoire: le Rickettsia conorii, infectant l'homme par l'intermédiaire d'un vecteur (tique brune du chien) Rhipicephalus sanguineus [1, 2] qui est à la fois vecteur et réservoir [3, 4]. Elle sévit dans le pourtour méditerranéen sur le mode endémique avec poussées épidémiques estivales. Son incidence sur le pourtour méditerranéen est évaluée à 50/100000 habitants /an. La fréquence au Maroc est encore mal connue, cependant, elle semble être fréquente sous nos climats [1]. Elle atteint de façon équivalente les deux sexes et toutes les tranches d'âge, avec une prédominance infantile et chez le sujet de plus de 60 ans [5]. Le diagnostic doit être évoqué devant un contexte épidémiologique compatible, une fièvre, une escarre (tache noire) au point d'inoculation et une éruption maculopapuleuse mais la confirmation du diagnostic se fait sur la sérologie (immunofluorescence directe avec séroconversion), la biologie moléculaire (notamment polymerase chain reaction (PCR) spécifique sur la biopsie de l'escarre ou comme récemment décrit sur l'écouvillon de l'escarre), ou la culture cellulaire des différents types de prélèvements [6]. La FBM est réputée d'évolution bénigne mais au cours des formes sévères de la maladie, des complications systémiques sont observées parmi lesquelles les signes neurologiques qui peuvent en faire parfois toute la gravité avec une fréquence estimée à 1% [2]. Les manifestations neurologiques habituellement décrites sont de sémiologie variable: méningite lymphocytaire, méningo-encéphalite, meningoencéphalomyélite aigue, myélite aigue, coma, polyradiculonévrite aigue, neuropathie périphérique, atteinte des nerfs crâniens [3] alors que les infarctus cérébraux liés à la rickettsiose ont été exceptionnellement rapportés dans la littérature [4, 7]. A notre connaissance, seulement quatre cas ont été rapportés et bien documentés. La physio-pathogénie des infarctus cérébraux dus à des agents infectieux est très variable; le mécanisme prépondérant est une vascularite par inflammation de la paroi artérielle en réponse à la présence du micro-organisme, avec réaction immune locale induisant des lésions de l'endothélium et la formation de thromboses vasculaires locales, voire la rupture des parois, expliquant les infarctus, les hémorragies voire la coexistence des deux types de lésions [8]. La prise en charge précoce raccourcit la durée des symptômes et empêche l'évolution défavorable. Le traitement de la vascularite à rickettsia dépend essentiellement d'une antibiothérapie adaptée permettant un contrôle de l'infection et par conséquent une réduction de la morbi-mortalité. Le traitement de référence est la doxycycline chez l'adulte (200 mg par jour) et chez l'enfant (5 mg/kg par jour). L'alternative pour les femmes enceintes et les enfants est la josamycine. La place des fluoroquinolones dans le traitement de la FBM constitue un sujet de débat, certains auteurs les préconisent en cas de formes graves surtout neurologiques, d'allergie ou de contre-indication aux cyclines, pendant une durée de sept jours [1, 2], d'autres préfèrent les éviter en se basant sur un travail récent montrant que le traitement par les Fluoroquinolones est associé à une évolution vers les formes sévères de la FBM [ 9, 10].

## Conclusion

Exceptionnellement rapporté dans le cadre des manifestations neurologiques des rickettsioses, l'infarctus cérébral reste une complication à ne pas méconnaitre surtout en regard d'un bilan étiologique en particulier cardiovasculaire demeuré négatif.
